# Rapid Production and Purification of Dye-Loaded Liposomes by Electrodialysis-Driven Depletion

**DOI:** 10.3390/membranes11060417

**Published:** 2021-05-31

**Authors:** Gamid Abatchev, Andrew Bogard, Zoe Hutchinson, Jason Ward, Daniel Fologea

**Affiliations:** 1 Department of Physics, Boise State University, Boise, ID 83725, USA; andybogard@u.boisestate.edu (A.B.); zoehutchinson@u.boisestate.edu (Z.H.); jasonward1@u.boisestate.edu (J.W.); 2 Biomolecular Sciences Graduate Program, Boise State University, Boise, ID 83725, USA

**Keywords:** liposomes, lipids, electrodialysis, lysenin, self-quenching

## Abstract

Liposomes are spherical-shaped vesicles that enclose an aqueous milieu surrounded by bilayer or multilayer membranes formed by self-assembly of lipid molecules. They are intensively exploited as either model membranes for fundamental studies or as vehicles for delivery of active substances in vivo and in vitro. Irrespective of the method adopted for production of loaded liposomes, obtaining the final purified product is often achieved by employing multiple, time consuming steps. To alleviate this problem, we propose a simplified approach for concomitant production and purification of loaded liposomes by exploiting the Electrodialysis-Driven Depletion of charged molecules from solutions. Our investigations show that electrically-driven migration of charged detergent and dye molecules from solutions that include natural or synthetic lipid mixtures leads to rapid self-assembly of loaded, purified liposomes, as inferred from microscopy and fluorescence spectroscopy assessments. In addition, the same procedure was successfully applied for incorporating PEGylated lipids into the membranes for the purpose of enabling long-circulation times needed for potential in vivo applications. Dynamic Light Scattering analyses and comparison of electrically-formed liposomes with liposomes produced by sonication or extrusion suggest potential use for numerous in vitro and in vivo applications.

## 1. Introduction

Liposomes are spherical vesicles that enclose an aqueous interior cavity protected by a unilamellar or multilamellar shell made of lipids, and the exploitation of their features has enabled the development of myriads of scientific and biomedical applications [1,2,3,4,5,6]. The liposome membrane is made of lipids commonly found in cell membranes, and their compositions may be adjusted by addition of specific lipids and sterols to better simulate the lipid partition of a particular membrane host. Such mimicking provides a simplified experimental system for surveying transport properties of membranes [4,7]. More fundamental exploration options are presented by the ability to reconstitute membrane receptors directly into membranes [8,9] or to functionalize the membrane surface by chemical addition of specific recognition elements [10,11,12]. Another important set of applications of liposomes originates in their ability to function as carriers for ions and molecules. Liposomes may transport hydrophilic, water-soluble cargo within their aqueous inner volume, and non-polar compounds embedded within the hydrophobic core of the membrane. These excellent transport capabilities led to the idea of using liposomes for transport and delivery of drugs to diseased organs and tissues in the human body [13]. The ability to adjust the physico-chemical properties of liposomes for drug delivery purposes is greatly exemplified by their FDA-approved clinical application for cancer therapy [14,15]. Liposome PEGylation significantly improves their circulation time by preventing recognition by the reticuloendothelial system (RES)/mononuclear phagocyte system (MPS) [16,17,18], while a small size enables self-accumulation into solid tumors by the Enhanced Permeability and Retention (EPR) effect [19,20,21]. Active loading of drugs such as doxorubicin enables the achievement of high local drug concentrations, greatly reducing systemic distribution by self-accumulating at the tumor site [22]. 

All production methods of liposomes used as carriers employ liposome preparation and loading. Irrespective of the production approach adopted, liposome preparation relies on the self-assembly of lipids, which is driven by their amphiphilic nature and interactions with water. Hydration from thin lipid films usually leads to the formation of multilamellar liposomes, which are further down-sized and rendered unilamellar by extrusion or sonication [23,24,25,26]. In this case, passive drug loading may be realized by direct addition to the hydrating solution, while active loading by electrochemical gradients may be achieved after liposome formation [27,28]. In a different approach, liposome preparation is achieved by further dilution of a solvent utilized to solubilize the lipids, which may be performed by organic solvent injection [29,30,31] or detergent removal [32,33,34,35,36]. These methods are well established, and each one has advantages and disadvantages with respect to equipment requirements, achievement of desired size, loading protocol and efficacy, and time. A major bottleneck common among multiple production methods is the time needed to complete the procedures and obtain loaded liposomes devoid of unloaded cargo in the bulk. 

To alleviate this problem, we propose producing loaded and purified liposomes by Electrodialysis-Driven Depletion (EDD) of detergent. Detergent removal has long been understood to create bilayers [33,37] and established as a method for preparing unilamellar liposomes [33,34,35,36,38]. Upon solubilizing lipids with a detergent, a mixed micelle formation consisting of detergent and lipids appear. Removal of the detergent results in fusion of micelles and bilayer disk formation. As the disk becomes larger, it will curve to minimize edge circumference to reduce hydrocarbon tail exposure to aqueous solution and eventually enclose to form a bilayer sphere, eliminating exposed edges [39,40]. 

Based on this body of evidence, we hypothesized that electrophoresis may lead to rapid depletion of ionic detergents from detergent-lipid mixtures and liposome formation. In addition, we anticipated that charged molecules intended as cargo may be trapped inside the formed liposomes before being cleared from the bulk by the action of the electric field. Our experimental results strongly support the applicability of EDD for fast liposome formation, loading, and purification. 

## 2. Materials and Methods

Asolectin (Aso, Sigma-Aldrich, St. Louis, MO, USA), cholesterol (Chol, Sigma-Aldrich, St. Louis, MO, USA), brain sphingomyelin (SM, Avanti Polar Lipids, Alabaster, AL, USA), 1,2-distearoyl-sn-glycero-3-phosphocholine (DSPC, Avanti Polar Lipids), and 1,2-distearoyl-sn-glycero-3-phosphoethanolamine-N-[methoxy(polyethyleneglycol)-2000] (DSPE-PEG, Avanti Polar Lipids) were purchased either in powder or chloroform-solubilized form. The powder lipids were solubilized in chloroform, mixed with the other lipids at the desired ratios in a glass vial, and had the solvent removed by being placed under vacuum for at least 12 h. Formed lipid films not used immediately were stored in a freezer at −20 °C. The precursors to all liposome preparations were the dried lipid films prepared from mixture of lipids at dry-weight ratios specified in the results section. KCl (ThermoFisher Scientific, Waltham, MA, USA), a stock solution of 1 M HEPES (Sigma-Aldrich) of pH 7.4, and cholic acid (CA, Thermo Scientific, Waltham, MA, USA) were used to prepare buffered ionic solutions (20 mM KCl, 5 mM Hepes) with or without addition of acridine orange (AO) or rhodamine 6G (R6G) (both from ThermoFisher Scientific) at 1 mM final concentration. The emission spectrum of each dye was determined with a FluoroMax4 spectrofluorometer (Horiba Scientific, Piscataway, NJ, USA) set in emission mode. The same instrument was used to establish the AO self-quenching plot, monitor AO and R6G migration under exposure to electric field, and measure the release kinetics of dyes loaded into liposomes. 

**Figure 1:**
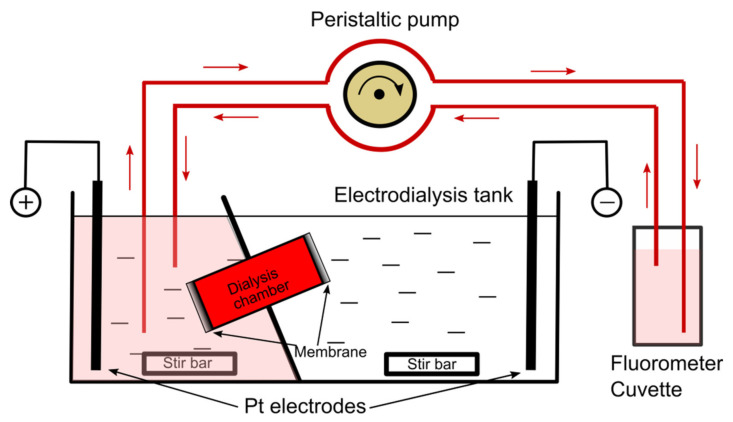
Experimental setup for liposome production by electrodialysis-driven depletion (EDD) and fluorescence monitoring. The custom setup includes the electrodialysis tank (ElectroPrep Electrodialysis System), an Ultra-Fast Dialyzer chamber, and a microfluidic setup to recirculate the solutions through a constant volume fluorometer cuvette for real-time fluorescence measurements. This specific setup describes migration and quantification of an anionic dye transferred from the dialysis chamber to the left reservoir. The solutions in the two reservoirs were continuously stirred with magnetic bars. The diagram is not to scale.

The electrodialysis-driven depletion (EDD) experiments employed a traditional use of the ElectroPrep Electrodialysis System (product #: 74-1196, Harvard Apparatus, Holliston, MA, USA), which was also adapted for real-time assessment of dye migration from the Ultra-Fast Dialyzer chamber (Harvard Apparatus) under exposure to electric fields in order to establish the time required to complete dye depletion from the chamber. The modified experimental setup (Figure 1) included the ElectroPrep Electrodialysis System completed with a custom fluidic system which continuously re-circulated the solution from either reservoir with a multi-port Gilson MiniPuls 3 peristaltic pump (Gilson Inc., Middleton, WI, USA) and fed a constant-volume flow cell (cuvette) for monitoring of fluorescence with the spectrofluorometer. Specific emission of each dye was measured in kinetics mode at 0.1 s integration time and a sampling rate of six samples/minute. The solution in the dialysis chamber mounted in the insulating separation wall was the only conducting pathway between the reservoirs. Consequently, charged molecules migrated from the dialysis chamber into the corresponding reservoir as dictated by the electrophoretic force. The Pt electrodes of the ElectroPrep Electrodialysis System were wired to a VWR Power Source 300 V electrophoresis power supply (VWR International, Radnor, PA, USA) set to constant current, and the solutions in the reservoirs were continuously stirred with magnetic stir bars. 

Liposome production by EDD comprised hydration and solubilization of lipid mixtures in 1 mL ionic solutions containing 2% (*w*/*v*) CA. To aid homogenization, the samples underwent a brief sonication (10 s) in a bath sonicator (Fisher Scientific), followed by 10 min heating at 75 °C and another brief sonication. After solubilization, the solutions were transferred into the double sided Ultra-Fast Dialyzer chamber equipped at both ends with polycarbonate membranes (10 nm pores, Harvard Apparatus), and mounted in the insulating separation wall of the ElectroPrep Electrodialysis tank filled with electrolyte solutions. 

Liposome preparation by sonication was performed with a Misonix S-4000 probe sonicator (Misonix, Farmingdale, NY, USA) equipped with a micro-tip. The lipid mixtures (with, and without dyes) were hydrated in warm electrolyte solutions, then placed into small glass vials and sonicated on ice for 15 min in manual mode at 25% amplitude, and a power transfer of 6–7 W. 

For extrusion, the lipid films in the glass vials were slowly hydrated for a few hours at 45 °C. To complete hydration and homogenize the mixture, the hydrated samples underwent four freeze/thaw cycles. The liposomes were extruded at 70 °C with an Avanti Polar Lipids extruder equipped with 200 nm polycarbonate filters for a total of 61 passes. 

Imaging was completed with an Olympus IX71 fluorescence microscope (Olympus Scientific Solutions Americas Corp, Waltham, MA, USA) equipped with filter sets specific for the used dyes. The release of encapsulated dyes was monitored from the fluorescence changes elicited by membrane permeabilization with 0.5% Triton X-100 (Sigma-Aldrich, St. Louis, MO, USA) [41,42]. A similar procedure was utilized to assess the unilamellarity of EDD-produced liposomes by inducing permeabilization with the pore-forming toxin lysenin (Sigma-Aldrich). 

For comparison between the three distinct production methods, liposomes were characterized by dynamic light scattering (DLS) with the Zetasizer Nano ZS (Malvern Panalytical Inc., Westborough, MA, USA) for determination of average hydrodynamic diameter and size distribution (PDI, polydispersity index) at room temperature. For each liposome sample we analyzed three sets, with each set consisting of 13 consecutive runs. Each set provided the corresponding average diameter and PDI, from which we calculated the mean values and standard deviations.

## 3. Results and Discussions

### 3.1. Dye separation by Electrodialysis

Successful separation by electrodialysis requires the detergent and dye molecules to possess an effective electric load. The used detergent (CA) is acidic (pKa = 4.8 [43]) and completely ionized near neutral pH; although many of the common fluorescent dyes are also charged for a large range of pHs, we did not know how long they would take to migrate from the dialysis chamber to the reservoirs upon exposure to an external electric field. A potential issue with the electrical conditions is that the electrical currents may lead to redox reactions at the electrodes (i.e., H^+^ and HO^−^ production, as well as other products that may also lead to undesired side reactions in solutions), and an increased temperature through the Joule effect. pH changes may affect the ionization status of the molecules and their migration, and they may also modulate the fluorescence of the dyes. To alleviate such potential issues, we sought to reduce the electrical currents in order to prevent major temperature and pH changes. While this may be simply realized by reducing the applied voltage, such an approach will also diminish the magnitude of the electrophoretic force, which may lengthen the time required for migration. After some experimentation, we established that the 20 mM KCl/5 mM HEPES (pH = 7.4) led to minor variations of temperature of the bulk ~2 °C) and pH in the dialysis chamber (~0.3 units) at 75 mA constant current applied for 45 min.

**Figure 2:**
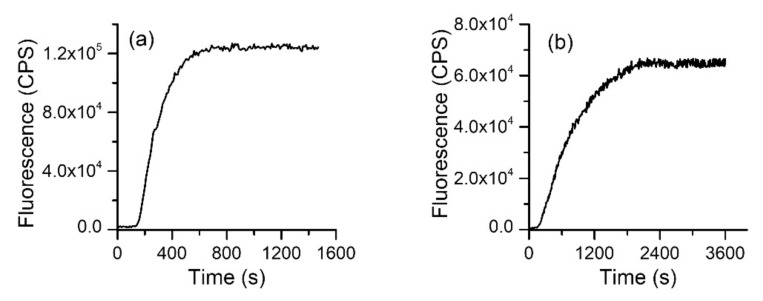
Electrodialysis leads to rapid depletion of charged dyes from solutions. Acridine orange (AO) (**a**) migrates faster than rhodamine 6G (R6G) (**b**) but both are depleted from the dialysis chamber and transferred into the reservoirs in less than 40 min.

To determine the time required for dye migration, we loaded the electrodialysis chamber with 1 mM of AO or R6G. The electrodialysis tank reservoirs were filled with dye-free ionic solutions and dye migration was estimated by employing the fluidic system described in the Materials and Methods section. The evolution of the fluorescence in reservoirs was estimated from kinetics measurements by employing the flow cell and the fluorometer. The wavelengths for excitation/emission were set apart for each of the dyes (AO: 485 nm/530 nm, R6G: 528 nm/550 nm), with a 1 nm slit for both excitation and emission. The power supply was set to 75 mA constant current, and the fluorescence measurements started ~10 s after applying the voltage. Both dyes started migrating from the chamber shortly after application of the electric field and the fluorescence monotonically increased until reaching a plateau indicative of migration completion (Figure 2). The kinetics profiles (i.e., the characteristic time) for the dyes were different, and AO migrated faster than R6G. However, the maximum signal was achieved in less than 40 min for both dyes, which set a reference time frame for further electrodialysis experiments. 

### 3.2. Simultaneous Liposome Formation, Loading and Purification by EDD

Detergent removal from the lipid-containing mixtures by dialysis drives the formation of self-enclosed structures [33,44]. We hypothesized that during enclosure, dye molecules can also be entrapped within the formed liposomes. Therefore, fast and concomitant liposome formation and loading may be achieved if the detergent and dye are electrophoretically driven outside the dialysis chamber.

**Figure 3:**
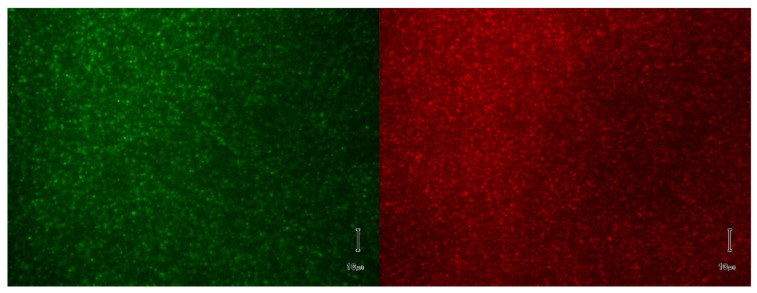
Microscopy imaging of fluorescent liposomes produced and purified by EDD. The liposomes are composed of asolectin and cholesterol and are loaded with AO (**left panel**) and R6G (**right panel**). The scale bar is 10 µm.

To test this hypothesis, we prepared lipid mixtures (Aso:Chol, 10:4 weight ratio) in ionic solutions containing 2% (*w*/*v*) CA to which 1 mM AO or R6G was added. The samples were subjected to electrodialysis at 75 mA constant current for up to 40 min. Figure 3 shows that simultaneous clearance of detergent and dye molecules leads to formation of loaded liposomes. 

### 3.3. Production of Long-Circulating Liposomes by Electrodialysis

**Figure 4:**
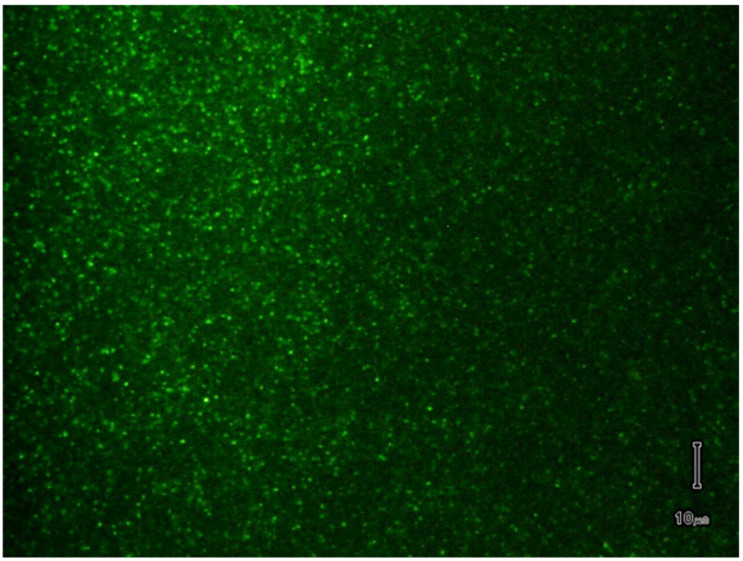
Microscopy image of PEGylated liposomes loaded with AO, prepared and purified by EDD. The scale bar is 10 µm.

The rapid clearance of liposomes from circulation constitutes a major roadblock for in vivo biomedical applications [45,46,47]. However, substantially improved circulation times are attained by adjusting the lipid composition of the membrane in order to minimize the undesired interactions with the defense system of the host. Addition of PEGylated lipids to the self-assembled membranes is often employed to extend the lifetime of liposomes in circulation, and such compositions are used for producing liposomes intended for cancer therapy and other in vivo applications [16,17,18,46,48,49]. To verify if electrodialysis is suitable for formation and loading of long-circulating liposomes, we prepared lipid mixtures containing DSPC, Chol, and DSPE-PEG (8.2:3.8:2.6 weight ratios). The lipids were solubilized in the buffered solution containing 2% CA and 1 mM AO, heated for 20 min at 75 °C, introduced into the Ultra-Fast Dialyzer chamber and subjected to a constant current of 75 mA for 20 min. Microscopy imaging revealed the formation of AO-loaded liposomes (Figure 4) and the good contrast ratio between liposomes and background suggested successful elimination of non-incorporated AO. 

### 3.4. Verification of Dye Loading 

There is no doubt that some of the dye present in the solubilization buffer is lost during the exposure to electrical currents due to migration before being trapped in the formed liposomes. To provide a rough estimation of the residual AO concentration inside PEGylated liposomes, we performed a release experiment that employed solubilization of liposomal membranes by addition of the non-ionic detergent Triton X-100 [41,42]. AO fluorescence presents self-quenching, i.e., a significant decrease in fluorescence manifested upon increase in dye concentration over 10 µM [50]. Although the exact mechanisms of self-quenching are not elucidated, it is considered that the intermolecular interactions occurring at high concentrations lead to a diminished fluorescence emission [50,51,52]. If self-quenching concentrations are attained inside liposomes, membrane solubilization leads to dye dissipation into the bulk, and the decrease in concentration over time is monitored from the increase in fluorescence [53].

**Figure 5:**
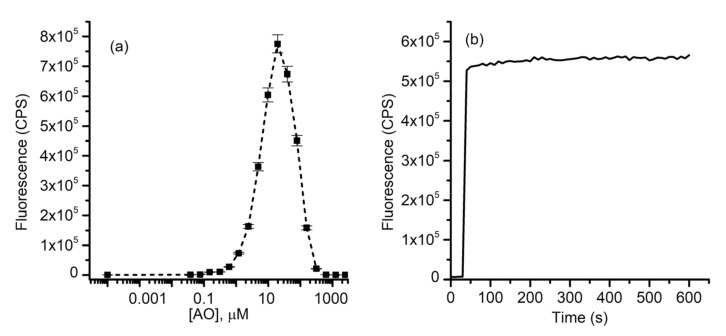
AO release from PEGylated liposomes produced, loaded and purified by EDD. (**a**) AO fluorescence indicates self-quenching at bulk concentrations over 10 µM. (**b**) The sustained release of AO from liposomes solubilized by addition of Triton X-100 indicates successful loading.

The changes in AO fluorescence intensity recorded upon addition of 100 µL of 5% Triton-X-100 to a 1.0 mL buffer solution containing 20 µL PEGylated liposomes produced by electrodialysis indicated that the AO concentration inside liposomes attained self-quenching levels (Figure 5). In addition, the fluorescence continually increased upon membrane solubilization, indicating that the AO concentration in the bulk did not fall below self-quenching level. 

### 3.5. Unilamellar or Multilamellar?

**Figure 6:**
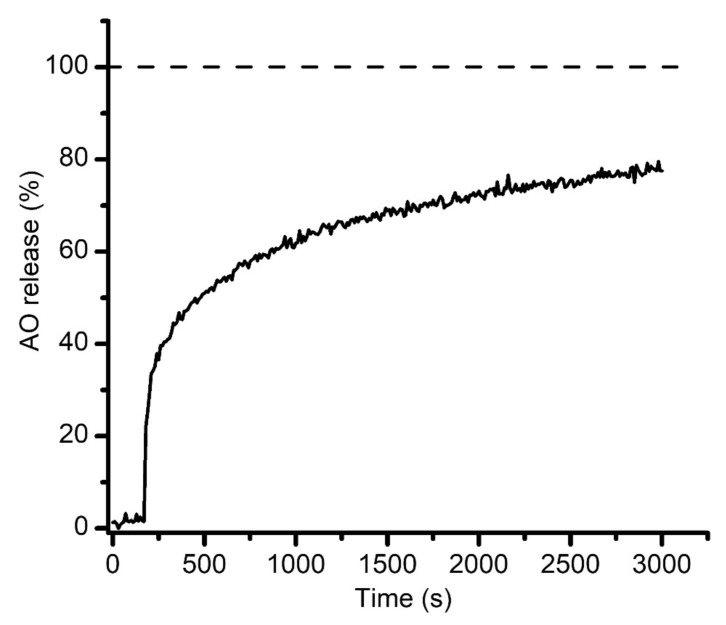
Lysenin-induced permeabilization of sphingomyelin-based liposomes. The sustained release of AO (~80% in less than one hour, relative to 100% release achieved by Triton X-100 addition) suggests that the target membranes are unilamellar. The dashed line shows the 100% release achieved by Triton X-100 addition.

Our next investigation addressed the lamellarity of the liposomes produced by EDD. Irrespective of the production method, a fraction of the liposomes will have the membrane consisting of multiple layers, which may impede their further application for purposes that require unilamellar liposomes. The fundamental difference between unilamellar and multilamellar liposomes is the number of lipid layers they consist of. Determining the number of layers in the membrane is not an easy task and may require sophisticated instruments and extensive preparatory tasks [54,55]. To answer this question, we proceeded with exploring the interactions between liposomal membranes and pore-forming toxins. This approach is based on the significant changes in the membrane permeability induced by the conductive pathways produced by the pore-forming toxins interacting with the target membranes; the leaky membrane leads to the release of incorporated dyes, which can be assessed by microscopy or fluorescence spectroscopy. For our investigations we used the prototype pore-forming toxin lysenin, which introduces large-conductance pores in artificial and natural membranes containing sphingomyelin [56,57,58,59]. However, for relevancy with regards to the membrane thickness, one may assume that lysenin may not span multiple bilayers [60], therefore the changes in membrane permeability are specific to unilamellar liposomes. Liposomes consisting of Aso, SM, and Chol (10:4:4 weight ratio) were produced and loaded with AO by electrodialysis as described in the previous sections and analyzed by fluorescence spectroscopy. The release of the dye was monitored from the changes in AO’s fluorescence upon addition of lysenin (~20 ng) to the cuvette containing 2 mL buffer and 100 µL liposomes. As inferred from the recorded kinetics (Figure 6), the release of the dye started immediately after lysenin addition, and monotonically increased for the total duration of the record (3000 s). As anticipated, the lysenin-induced release was slower than the detergent-induced release since lysenin channels must first interact with the target membranes and oligomerize into functional pores to induce release of the dye. The fluorescence asymptotically approached a steady state, which corresponds to ~80% of the total release induced by Triton X-100 addition (Figure 6). 

This experiment suggests that most of the target membranes are unilamellar; nonetheless, this is not irrefutable proof that all the membranes are solely consisting of lipid bilayers. Unilamellar and multilamellar patches may be present within the same liposomes, and the unilamellar portion of the membrane may facilitate lysenin-induced permeabilization and dye release. 

### 3.6. EDD Comparison to Extrusion and Sonication 

**Figure 7:**
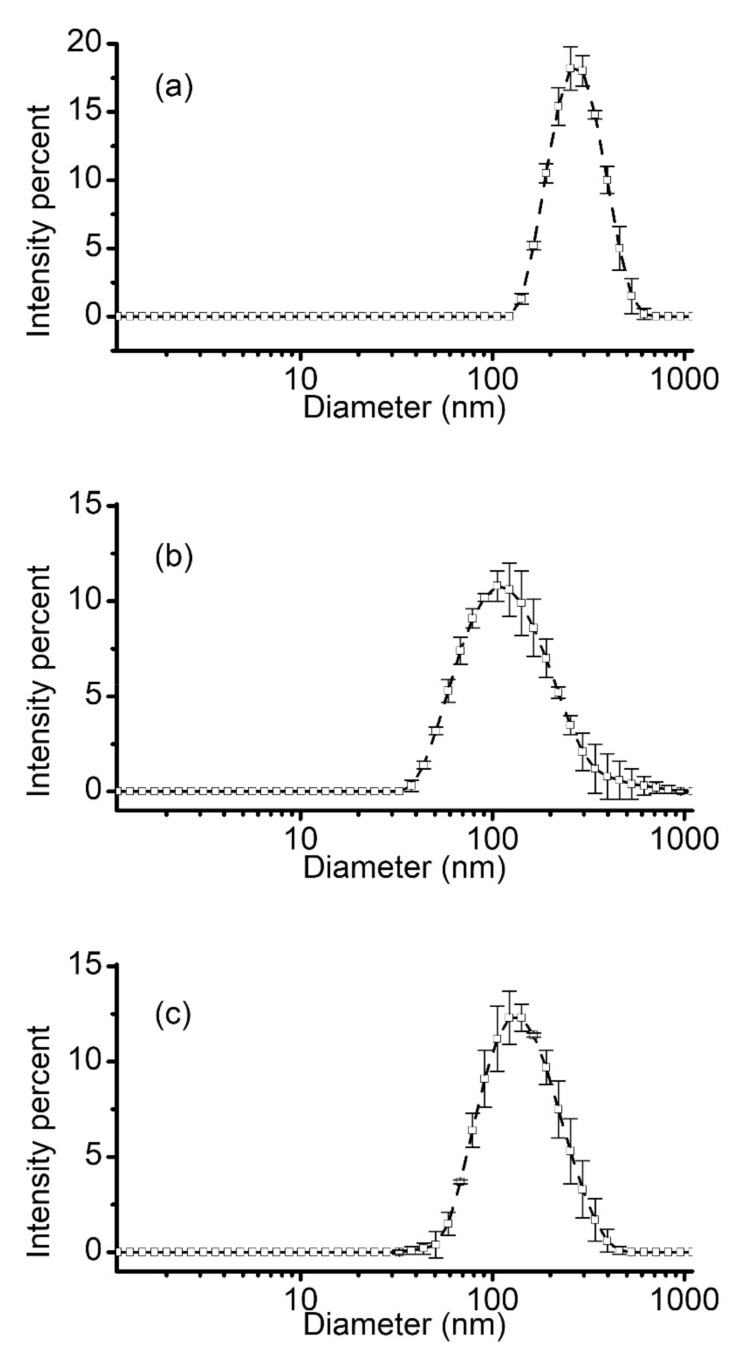
Dynamic light scatterring characterization of liposomes produced by extrusion (**a**), probe sonication (**b**), and EDD (**c**). Each plot shows the mean intensity percent ± SD (n = 3) determined as a function of diameter.

Two well-established methods of liposome preparation are extrusion and sonication [23], which have been widely and successfully used for decades [61]. Extrusion refines liposomes formed by hydration and self-assembly to render them unilamellar and adjust their size by passage through membrane filter pores of a particular size [25,26]. Sonication also generates relatively consistent and evenly distributed populations of unilamellar liposomes in a short period of time with low effort, although their size is not easily controlled. However, production of loaded liposomes by either method requires further purification steps to remove the unincorporated molecules from bulk. When charged cargo is used for liposome loading, EDD may eliminate the necessity of further purification and significantly reduce the time required for preparation of loaded liposomes. To further assess the quality of liposomes prepared by the three different methods (extrusion, sonication, and EDD), we compared their physical characteristics by DLS. The three experiments utilized identical lipid compositions (10:4 mass ratio of Aso to Chol) and ionic solutions; only electrodialysis comprised addition of CA for solubilization. For consistency, all the lipids were first mixed in chloroform, placed in glass vials, vacuumed overnight for solvent removal, and the formed thin films were hydrated for 2 h at 45 °C. Liposomes were prepared by extrusion, sonication, and EDD as described in the methods section. DLS analysis (Figure 7) indicated that extrusion provided an average diameter of 259.6 ± 2.4 nm and the narrowest size distribution with a PDI of 0.077 ± 0.029. Liposomes obtained by sonication presented a significantly smaller average diameter of 114.2 ± 1.8 nm with a larger size distribution, having a PDI of 0.250 ± 0.013. EDD led to formation of liposomes characterized by an intermediate size, with an average diameter of 134.8 ± 0.7 nm and a PDI of 0.214 ± 0.011.

A simple comparison between the three methods shows the most uniform size distribution is achieved by extrusion. This method also enables controlling the average diameter of the liposomes by choosing appropriate membrane filters, which are available in a large range of pore sizes. Both sonication and EDD are fast, simple, and provide satisfactory size distribution of produced liposomes. In both cases however, the size of the produced liposomes is not easily adjusted from experimental conditions. 

The physical characteristics of the EDD-produced liposomes together with the ability to utilize lipid compositions that improve their circulation time suggest that they are suitable for a large variety of scientific and biomedical applications [62]. An advantage of the EDD method over others is its ability to simultaneously form, load, and separate liposomes from the non-incorporated cargo, therefore significantly reducing the time needed for purification. However, beside the necessity of using charged detergents and dyes, liposome production by EDD has other potential limitations. Changes in pH and solution compositions during electrodialysis may alter the physical and chemical properties of the molecules (i.e., ionization state, or fluorescence). This is particularly concerning if the cargo molecules are heavily reliant on such properties for their intended purpose and efficacy. A simple solution to address this problem was the use of a low ionic strength electrolyte solution. Such solutions may lead to osmotic balance issues, but they may be mitigated by including neutral molecules (i.e., sugars) in the solutions to ensure iso-osmolarity. Although EDD is similar to other solvent-removal methods (including detergent removal by simple dialysis), we do not have an estimate of the amount of detergent left in solutions or membranes. We successfully tested several lipid/dye compositions but one universal setting for successful EDD might be elusive. Therefore, pretreatment conditions, such as temperature and solution agitation, as well as solution and electrical conditions may need to be tailored to other lipids and cargo used for EDD preparation of loaded liposomes. 

## 4. Conclusions

In summary, EDD may be employed for fast and cost-effective production of loaded and purified liposomes. The size and distribution quality of the liposomes attainable with EDD are comparable to extrusion and sonication. Further investigations of the various settings and parameters and their influence on liposome formation and loading may provide a better understanding of the limitations and full potential presented by this method for scientific and biomedical applications.

## Data Availability

Not applicable.
